# Unveiling the power of social influence: how functional, emotional, and social values drive Pilates participation through an extended TPB model

**DOI:** 10.3389/fpsyg.2025.1453874

**Published:** 2025-02-28

**Authors:** Sukkyu Kim, Yunduk Jeong, Youngsu Jung

**Affiliations:** ^1^Dongguk University WISE, Gyeongju, Republic of Korea; ^2^Kookmin University, Seoul, Republic of Korea; ^3^Dong-A University, Busan, Republic of Korea

**Keywords:** Pilates, perceived value, theory of planned behavior, behavioral intention, subjective norms, social influence

## Abstract

**Purpose:**

This study investigates how functional, emotional, and social values shape Pilates participants’ attitudes and behavioral intentions within an Extended Theory of Planned Behavior (ETPB) framework.

**Method:**

A survey was conducted with 288 Pilates participants, and structural equation modeling was employed to analyze the relationships among perceived value, attitude, subjective norms, perceived behavioral control, and behavioral intention.

**Results:**

The findings indicate that functional and social values positively influence attitudes, whereas emotional value has no significant effect. Moreover, subjective norms exert the strongest influence on behavioral intention, highlighting the importance of social factors in Pilates participation.

**Discussion/conclusion:**

This study underscores the role of perceived value in shaping attitudes and confirms the dominant impact of subjective norms on behavioral intention. The findings offer theoretical contributions by refining the ETPB model and provide practical insights for enhancing engagement in Pilates programs.

**Originality/value:**

This study contributes to the literature by applying an ETPB model to the context of Pilates, incorporating perceived value as an additional factor. It challenges the traditional emphasis on attitude as the primary predictor of behavioral intention by highlighting the dominant role of subjective norms. These insights offer a more nuanced understanding of the factors driving Pilates participation and provide practical implications for developing strategies to enhance engagement and retention in Pilates programs.

## Introduction

*‘Pilates is not just exercise; it is a pathway to a balanced and harmonious body and mind’* (Kim and Jeong, 2022).

Pilates, originally developed in the 1920s, has become a globally recognized exercise method known for enhancing physical health and well-being (Joseph and Simona, 2004). Given its increasing popularity, it is crucial to understand the factors influencing participation. In South Korea, Pilates ranks among the most popular fitness activities, following golf and swimming, with health maintenance being the primary motivation for participation. The growing preference for Pilates can be attributed to its promotion through various media as a health and fitness program, leading to its perception as a viable option for daily exercise. Consequently, the participation rate in Pilates has been steadily increasing, and the number of related facilities has grown to meet the demand (Jang and Kim, 2019).

Perceived value plays a pivotal role in shaping consumer attitudes and behaviors (Zeithaml, 1988; Kim and Ryu, 2016). While prior studies have explored its role in various domains, research on its impact within the context of exercise participation, particularly Pilates, remains limited. This study addresses this gap by examining how different dimensions of perceived value influence attitudes and behavioral intentions in Pilates participation.

Various theories have been developed to understand and predict human behaviors. The Theory of Planned Behavior (TPB), proposed by Ajzen (1991), is a prominent theory in this regard, particularly in explaining exercise behavior at the decision-making level. Since Pilates participation is a voluntary exercise behavior, it aligns well with the TPB framework. The decision to engage in Pilates can be influenced by individuals’ attitudes toward Pilates, perceived social norms, and their perceived ability to continue practicing it.

However, previous studies indicate that the TPB alone may not adequately encompass the intricacies of decisions regarding exercise participation. The TPB elucidates intention development via attitude, subjective norms, and perceived behavioral control; yet, supplementary elements, such as perceived value, can substantially affect individuals’ motivation to engage in fitness activities (Kim and Moon, 2015). This study used an extended TPB (ETPB) model by integrating perceived value as an extra predictor, hence offering a more thorough comprehension of Pilates participation behavior.

Building upon this theoretical foundation, the TPB incorporates the concept of perceived behavioral control (PBC) into the Theory of Reasoned Action (TRA) to better predict individuals’ behaviors and intentions. PBC refers to an individual’s perceived ease or difficulty in performing a specific behavior (Ajzen, 1991), based on the perceived presence of factors that may impede or facilitate the behavior and the perceived power of these factors (Ajzen, 1991; Han et al., 2014). Incorporating PBC into behavior analysis has enhanced the predictive power for individual decision-making (Armitage and Conner, 2001; Ajzen and Fishbein, 2000).

In this theoretical context, Hagger et al. (2002) applied the TPB to predict exercise behavior, finding that attitudes toward exercise, subjective norms, and perceived behavioral control significantly influenced behavioral intention. Similar results were reported in studies predicting participation intention and actual participation in fitness exercises among female participants (Kwon et al., 2017) and continuous exercise participation among college students in physical education courses (Seo et al., 2015). These studies confirm that the TPB provides a robust framework for explaining behavioral intentions and exercise behaviors (Hausenblas et al., 1997).

While TPB has been widely applied in exercise research, its application to Pilates remains limited. This study addresses this gap by extending the TPB model to analyze Pilates participation. Unlike previous studies that primarily focused on general fitness or other types of exercise, this research specifically investigates the effects of functional, emotional, and social values on attitudes toward Pilates. Moreover, it evaluates how these attitudes, along with subjective norms and perceived behavioral control, influence behavioral intentions and continuous participation intentions for Pilates. This study’s primary contribution lies in its novel application of the TPB to the context of Pilates, providing insights that are specific to this increasingly popular form of exercise. By incorporating the concepts of functional, emotional, and social values, this research offers a more comprehensive understanding of the motivational factors influencing Pilates participation. Additionally, the findings are expected to inform strategies for fitness professionals and facility managers to enhance engagement and retention among Pilates participants. By developing a behavior model that predicts Pilates students’ participation intentions and behaviors, this study contributes to the broader literature on exercise psychology and consumer behavior.

Despite the increasing popularity of Pilates as a fitness activity, empirical research on the psychological and behavioral determinants of Pilates participation remains limited (Kim and Jeong, 2022). While existing studies have extensively examined general exercise behaviors using the TPB framework (Lu et al., 2022; Zhang et al., 2022), few have specifically explored how perceived value influences Pilates engagement. Considering that Pilates prioritizes accuracy, control, and mind–body coordination—unlike traditional fitness programs—its motivating factors for participation may vary from those of typical workout activities. Comprehending these distinct characteristics is crucial for formulating focused ways to improve engagement and retention in Pilates programs. This gap highlights the necessity for additional research on how perceived value influences attitudes and behavioral intentions within the realm of Pilates.

Therefore, this study aims to examine the major values and participation intentions of Pilates students using an extended model of the Theory of Planned Behavior (TPB). Specifically, this study evaluates the effects of functional, emotional, and social value on attitudes toward Pilates and the impact of attitude, subjective norms, and perceived behavioral control on behavioral intention and continuous participation intention in Pilates. The findings are expected to inform strategies for guiding individuals to make rational decisions by developing a behavioral model predicting Pilates students’ behavior.

## Theoretical framework and research hypotheses

### Perceived value

Value can be defined as consumers’ overall evaluation of a product (Zeithaml, 1988). Value, developed from the benefits gained from a product and the cost related to these benefits, leads to a positive evaluation of the company in the long run. The value types can be categorized based on three dichotomous dimensions: self-oriented or other-oriented, active or reactive, and intrinsic or extrinsic (Holbrook, 1999). Consumers’ perceived value is the fourth most important factor in attracting consumers, following quality, satisfaction, and loyalty (Jamal and Muhammad, 2011).

Perceived value is a multifaceted concept that includes functional, emotional, and social dimensions, which profoundly influence customer attitudes and behavioral intentions (Sweeney and Soutar, 2001). Recent studies have expanded the application of perceived value in exercise psychology. For instance, Kim and Moon (2015) examined how perceived value influences individuals’ motivation to engage in fitness activities, highlighting its significant role in shaping exercise intentions. Similarly, Ha and Kim (2023) analyzed the behavioral intention of Pilates participants by applying an extended theory of planned behavior (ETPB) model, incorporating perceived value as a key factor. These findings support the integration of perceived value within the ETPB framework for understanding Pilates participation.

Previous studies show that perceived value is essential in shaping favorable attitudes towards exercise behavior, therefore affecting intentions to participate (Kim and Moon, 2015). Functional value denotes the tangible advantages of an activity, including the promotion of physical health and the development of skills. Emotional value encompasses the inherent pleasure and psychological fulfillment obtained from engagement. Social value denotes the relational and societal advantages linked to group-oriented activities (Sheth et al., 1991). This study used an Extended TPB model, asserting that functional, emotional, and social values influence the development of attitudes about Pilates involvement. Sheth et al. (1991) expanded the concept of perceived value by presenting five elements: social, emotional, functional, conditional, and epistemic. In this study, a multidimensional approach was taken to investigate perceived value, focusing on the analysis of perceived value based on the study by Sweeney et al. (1999), which categorized value into social, functional, and emotional dimensions.

The TPB posits that attitude is a key determinant of behavioral intention (Ajzen, 1991). While TPB traditionally emphasizes cognitive evaluations in shaping attitudes, recent studies have highlighted the importance of perceived value in this process (Sweeney and Soutar, 2001; Kim and Moon, 2015). Perceived value encompasses functional, emotional, and social aspects, which influence attitudes toward behavioral engagement. Accordingly, this study integrates the perceived value framework into TPB, hypothesizing that functional, emotional, and social value positively influence attitudes toward Pilates participation.

Similarly, Teng et al. (2014) assessed the intention to visit an eco-friendly restaurant using the VAB model, reporting that higher perceived value leads to a higher level of positive attitude, which in turn is associated with a stronger behavioral intention and visit intention. They also claimed that attitude mediates the causal relationship between perceived value and behavioral intention.

As described above, previous studies on the influence of perceived value on attitude suggest that perceived value is a variable applicable as an indicator for predicting potential customer behavior (Bae et al., 2015). Therefore, this study examined which values positively affect attitudes among Pilates participants by applying a multidimensional concept of perceived value. Additionally, the study investigated the influence of the attitudes formed through perceived value on the behavioral intention of Pilates participants by applying a model of the extended ETPB.

*H1. Pilates participants’ functional value will have a significant positive effect on their attitude toward Pilates.*

*H2. Pilates participants’ emotional value will have a significant positive effect on their attitude toward Pilates.*

*H3. Pilates participants’ social value will have a significant positive effect on their attitude toward Pilates.*

### Theory of planned behavior (TPB) and extended theory of planned behavior (ETPB)

Fishbein and Ajzen (1975) proposed the Theory of Planned Behavior (TPB), an extension of the Theory of Reasoned Action (TRA). The TPB suggests that behavioral intention and behavior prediction depend on three key factors: attitude toward the behavior, subjective norms, and perceived behavioral control (Ahmed et al., 2017). Attitude refers to positive or negative evaluations of performing the behavior (Seo and Lee, 2015), while subjective norms encompass social influences and represent the general views of significant others about the behavior (Cho, 2019). Perceived behavioral control relates to an individual’s confidence or ability to control the necessary factors for performing a particular behavior, essentially reflecting the perceived ease or difficulty of performing the behavior (Ajzen, 1991; Kim, 2014). The TPB posits that these three factors influence an individual’s intention to perform a behavior, which subsequently impacts the actual execution of the behavior (Taylor and Todd, 1995).

Ajzen (1991) also suggested that additional predictor variables could be included if they enhance the TPB’s explanatory power. While the TPB is generally considered superior to existing behavioral theories in predicting behavioral intentions and actual behaviors (Dawkins and Frass, 2005; Lam and Hsu, 2004), some researchers argue that its explanatory power can still be insufficient. Therefore, they recommend adding antecedents that can improve the theoretical model’s explanatory power (Bagozzi, 1981; Ouellette and Wood, 1998; Vallerand et al., 1993).

As a result, studies have emerged attempting to apply an ETPB model by incorporating additional variables into the TPB framework. For instance, Yoon et al. (2010) included perceived risk and prior knowledge to investigate Koreans’ intention for international travel. Quintal et al. (2010) added perceived risk and uncertainty in their analysis. Lin and Chen (2006) incorporated prior knowledge, finding that it positively affects a consumer’s purchase decision and decision-making process for choosing a brand, strongly impacting behavioral intention. Kim and Moon (2015) examined the influence of value on the behavioral intention for outdoor recreational activities among automobile campers.

Building on these previous studies, this research examines the influence of perceived value on the behavioral intention of Pilates participants through a conceptual extension of the TPB. Specifically, it considers perceived value as an additional variable alongside attitude, subjective norms, and perceived behavioral control, aiming to explore how these factors collectively influence Pilates participants’ behavioral intentions.

**Figure 1:**
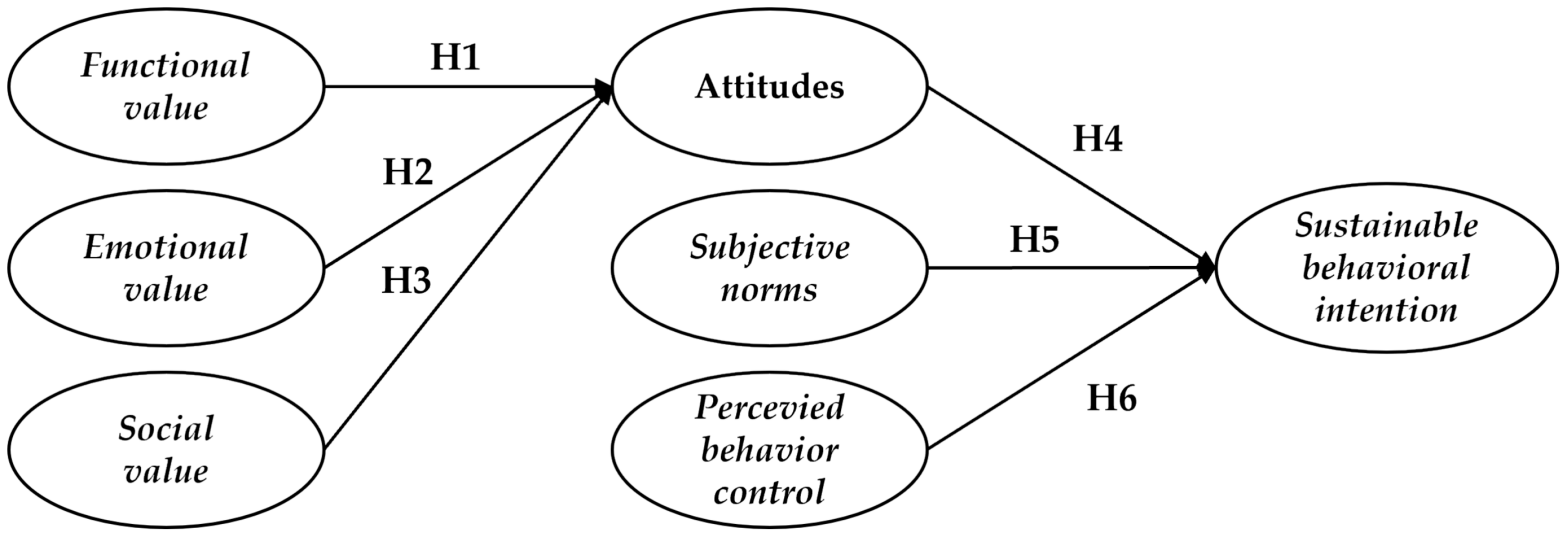
Conceptual model.

Based on the theoretical framework, the following hypotheses were formulated: (See Figure 1).

*H4. Pilates participants’ attitudes will have a significant effect on their behavioral intention for Pilates.*

*H5. Pilates participants’ subjective norms will have a significant effect on their behavioral intention for Pilates.*

*H6. Pilates participants’ perceived behavioral control will have a significant effect on their behavioral intention for Pilates.*

A summary of the proposed hypotheses and their supporting literature is presented in Table 1.

**Table 1:** Summary of hypotheses.

Hypothesis	Relationship
H1	Functional value → Attitude
H2	Emotional value → Attitude
H3	Social value → Attitude
H4	Attitude → Behavioral intention
H5	Subjective norms → Behavioral intention
H6	Perceived behavioral control → Behavioral intention

This study expands previous studies by exploring the influence of perceived value on Pilates participants’ behavioral intentions through an extended TPB model. Perceived value is examined as an additional determinant alongside attitude, subjective norms, and perceived behavioral control to understand how these factors collectively shape Pilates engagement. While TPB has been widely applied in various fitness contexts—such as general exercise adherence (Lu et al., 2022; Zhang et al., 2022) and recreational sports participation (Kim and Kim, 2021)—limited studies have specifically investigated its relevance to Pilates. Unlike conventional fitness programs, Pilates emphasizes controlled movements, posture alignment, and mind–body integration, which may amplify the role of perceived value in forming participation attitudes. Given these unique characteristics, incorporating perceived value into the TPB framework provides a more comprehensive perspective on Pilates participation.

In summary, this study seeks to understand the relationships between perceived value, attitudes, subjective norms, perceived behavioral control, and the behavioral intentions of Pilates participants by extending the TPB framework. The findings are expected to provide valuable insights into the factors influencing Pilates participation, thereby informing strategies to enhance engagement and retention in Pilates programs.

## Method

### Data collection

Data was collected through a questionnaire survey conducted during personal on-site visits among Pilates participants. The questionnaire was administered in person using a paper-based survey. The participants were selected using convenience sampling, a non-probability sampling method. The target population comprised Pilates students enrolled in Pilates centers located in Busan, Ulsan, and Gyeongbuk Province. The participants were selected using convenience sampling, a non-probability sampling method. The target population comprised individuals participating in Pilates classes in South Korea. Although the exact size of the total Pilates population is unknown, according to the 2021 data from Statistics Korea, the participation rate for yoga, taebo, and Pilates combined was approximately 7.2% among those engaged in sports activities. A total of 301 questionnaires were distributed, and participants were asked to complete the questionnaire using a self-administered survey method. After collection, 288 questionnaires were deemed suitable for analysis, as 13 questionnaires were excluded due to issues such as insincere or duplicate responses. Table 2 provides the general characteristics of the participants.

**Table 2:** Characteristics of subjects.

	Characteristics	*N* (%)
Gender	Male	38 (13.1)
	Female	250 (86.8)
Ages	10’s	15 (0.5)
	20’s	104 (36.1)
	30’s	112 (38.8)
	40+ years old	57 (19.7)
Careers	Less than 6 months	61 (21.1)
	6 months – less than 1 year	74 (24.6)
	More than 1 year – Less than 2 years	62 (21.5)
	More than 2 years – Less than 3 years	59 (20.4)
	More than 3 years	32 (11.1)

### Ethical considerations

This study adhered to ethical research guidelines, ensuring voluntary participation, anonymity, and data confidentiality. Participants were informed about the purpose of the study, and their consent was obtained before completing the survey. No personally identifiable information was collected, and all responses remained anonymous.

### Measure

In this study, a questionnaire survey was conducted to collect data. Except for questions regarding the general characteristics of participants, each item on functional, emotional, and social values, subjective norms, perceived behavioral control, and behavioral intention was rated on a five-point Likert scale ranging from ‘Strongly disagree/Not at all’ to ‘Strongly agree/Extremely.’ The scale for perceived value, which explains attitudes toward behavior and is composed of the three dimensions of functional, emotional, and social values, was adapted from Sweeney and Soutar (2001). The original scale was modified and complemented for this study, incorporating elements from Yoon et al. (2011). The final scale to assess perceived value consisted of 11 items: four items on functional value, four items on emotional value, and three items on social value. For the TPB constructs, a modified version of the scale developed by Ajzen and Driver (1992) and Ajzen (2001) was used. This scale was adjusted and complemented to suit the purpose of this study, based on the modifications suggested by So (2021). The TPB scale used in this study consisted of 16 items: five items on attitude, four items on subjective norms, three items on perceived behavioral control, and four items on behavioral intention. Table 3 presents the detailed survey measurement items, including the number of items per construct and their sources.

**Table 3:** Summarized survey measurement items.

Construct		Example survey item
Functional value	1	Pilates helps me improve my physical strength.
	2	Pilates improves my flexibility and balance.
	3	Pilates enhances my overall physical condition.
	4	Pilates is beneficial for my daily activities.
Emotional value	1	I feel happy and satisfied after doing Pilates.
	2	Pilates helps me relieve stress.
	3	Pilates gives me a sense of accomplishment.
	4	Pilates participation makes me emotionally fulfilled.
Social value	1	I enjoy doing Pilates because I can interact with others.
	2	Pilates participation strengthens my social relationships.
	3	Pilates makes me feel part of a community.
Attitude	1	I have a positive attitude toward participating in Pilates.
	2	Pilates is an enjoyable activity.
	3	I believe that doing Pilates is beneficial for me.
	4	I am satisfied with my decision to do Pilates.
	5	I consider Pilates as an important part of my lifestyle.
Subjective norms	1	People who are important to me think I should do Pilates.
	2	My family and friends encourage me to do Pilates.
	3	Many people around me think Pilates is a good activity.
	4	My peers influence my decision to participate in Pilates.
Perceived behavioral control	1	I believe I can consistently participate in Pilates.
	2	I have the necessary resources to continue doing Pilates.
	3	I can overcome any obstacles that might prevent me from doing Pilates.
Behavioral intention	1	I intend to continue doing Pilates in the future.
	2	I plan to regularly attend Pilates sessions.
	3	I will recommend Pilates to my friends.
	4	I will make an effort to prioritize Pilates in my schedule.

To ensure content validity, the modified survey items were reviewed by a physical education professor and two researchers holding PhDs in physical education. The experts evaluated if the modified items accurately measured the desired constructs related to Pilates participation. In response to their suggestions, slight adjustments were implemented to enhance clarity and relevance prior to finishing the questionnaire. Prior to distributing the primary survey, a pre-test was administered to a small cohort of Pilates participants (*N* = 77) to evaluate the clarity and comprehensibility of the revised items. Minor phrasing tweaks were implemented to improve clarity based on their suggestions. The finished questionnaire was prepared for data collection.

### Statistical analysis

In this study, 288 completed questionnaires were selected as valid samples for data analysis. The analysis was carried out using SPSS 22.0 and AMOS 24.0 software. First, frequency analysis and descriptive statistics were performed to examine the demographic characteristics of the participants. This step provided an overview of the sample’s demographic profile, including age, gender, and other relevant attributes. Next, reliability analysis was conducted using Cronbach’s *α* coefficient to assess the internal consistency of the scales. This ensured that the items within each scale were consistently measuring the same underlying construct. To assess the convergent validity of the measurement models, confirmatory factor analysis (CFA) was conducted using AMOS 24.0. This analysis verified that the items used in the scales adequately represented the underlying constructs they were intended to measure. Correlation analysis was then performed to test for multicollinearity among the variables. This step was crucial to ensure that the variables included in the model were sufficiently independent of each other. Finally, structural equation modeling (SEM) was employed to examine the causal relationships between the measured variables. SEM allowed for a comprehensive analysis of the hypothesized model, enabling the assessment of both direct and indirect effects among the constructs.

### Confirmatory factor analysis and reliability analysis

Using the collected questionnaires as analysis data, confirmatory factor analysis (CFA) was conducted to determine if the internal structure of each variable was adequately constructed. This analysis was essential to confirm that the items were measuring the intended constructs properly. In addition to CFA, reliability analysis was performed using Cronbach’s *α* coefficient to assess both construct validity and reliability. This step ensured that the measurement tools were consistently reliable and valid.

For model fit indices, the Comparative Fit Index (CFI), Tucker-Lewis Index (TLI), and Root Mean Square Error of Approximation (RMSEA) were applied. These indices were chosen because they are relatively insensitive to sample size and consider model simplicity (Hair et al., 2010). The thresholds for model fit indices were set as follows: CFI and TLI values of 0.90 or higher, and RMSEA values of 0.08 or lower (Hu and Bentler, 1999; Kline, 2011). All threshold conditions were satisfied in this study. Furthermore, for each factor, the Average Variance Extracted (AVE) value was 0.6 or higher, indicating adequate convergent validity. The construct reliability and Cronbach’s α coefficient were both 0.7 or higher, surpassing the threshold for reliability (Hair et al., 2010). These results confirm that the constructs measured in this study are both valid and reliable. Table 4 provides the detailed results of the above analyses.

**Table 4:** Summarized results for the validity and reliability assessments.

Variable		Items	Standardization factor	Concept trust	AVE	α
Perceived value	Functional value (FV)	1	0.784	0.894	0.680	0.893
		2	0.881			
		3	0.830			
		4	0.802			
	Emotional value (EV)	5	0.710	0.906	0.709	0.911
		6	0.796			
		7	0.901			
		8	0.942			
	Social value (SV)	9	0.761	0.834	0.628	0.833
		10	0.787			
		11	0.828			
Theory of behavior	Attitudes (AT)	1	0.844	0.937	0.751	0.924
		2	0.923			
		3	0.838			
		4	0.900			
		5	0.826			
	Subjective norms (SN)	6	0.870	0.951	0.829	0.951
		7	0.910			
		8	0.909			
		9	0.952			
	Perceived behavioral control (PBC)	10	0.741	0.860	0.674	0.770
		11	0.932			
		12	0.778			
Sustainable Behavioral Intention (SBI)		1	0.889	0.962	0.864	0.967
		2	0.849			
		3	0.972			
		4	1.002			

χ^2^ = 651.024, df = 301, CFI = 0.959, TLI = 0.953, RMSEA = 0.058.

Discriminant validity was further assessed using the Fornell-Larcker criterion and the Heterotrait-Monotrait (HTMT) ratio. Table 5 presents the Fornell-Larcker results, demonstrating that the square root of AVE values for each construct exceeded their corresponding inter-construct correlation coefficients, confirming discriminant validity. Furthermore, Table 6 reports the HTMT ratios, all of which were below the conservative threshold of 0.85 (Henseler et al., 2015), further verifying discriminant validity. These results indicate that the constructs in this study are distinct and measure separate theoretical concepts.

**Table 5:** Discriminant validity.

	FV	EV	SV	ATT	SN	PBC	SBI
FV	**0.82**						
EV	0.517**	**0.85**					
SV	0.576**	0.646**	**0.81**				
ATT	0.235**	0.287**	0.134**	**0.87**			
SN	0.165**	0.169**	0.067**	0.763**	**0.88**		
PBC	0.226**	0.185**	0.190**	0.604**	0.503**	**0.82**	
SBI	0.010**	−0.037**	−0.113**	0.029**	0.130**	0.125**	**0.93**

**p < 0.01. The bold values in table represent the square root of the average variance extracted (AVE), which is used to assess discriminant validity.

**Table 6:** Fornell-Larcker criterion for discriminant validity.

	FV	EV	SV	ATT	SN	PBC	SBI
FV	1.00						
EV	0.67	1.00					
SV	0.72	0.75	1.00				
ATT	0.31	0.37	0.22	1.00			
SN	0.22	0.25	0.15	0.85	1.00		
PBC	0.27	0.22	0.21	0.65	0.55	1.00	
SBI	0.02	−0.05	−0.14	0.03	0.17	0.15	1.00

In addition to correlation analysis, Variance Inflation Factor (VIF) values were computed to evaluate multicollinearity among predictor variables. The findings indicated that all VIF values varied from 1.98 to 2.74, much below the established threshold of 10 (Thompson et al., 2017), hence demonstrating the absence of multicollinearity concerns.

## Results

### Correlation analysis

Correlation analysis is a valuable tool for hypothesis verification in exploratory research, as it provides a broad overview of the relationships between variables. By showing the strength of the relationships between key variables, correlation analysis helps to outline the connections used in all research hypotheses before performing detailed hypothesis testing. In this study, Pearson’s correlation analysis was conducted to examine the correlations and directionality for each scale related to each research unit. Table 5 presents the results of this analysis. The analysis results revealed that the relationships between variables aligned with the hypotheses formulated in this study. Additionally, all correlation coefficients were lower than 0.80, indicating that multicollinearity was not a concern. The results of the correlation analysis suggest that the variables are sufficiently independent to be included in the subsequent structural equation modeling (SEM) analysis, thereby supporting the validity of the constructs used in this study.

### Tests of model fit for the research model

Based on previous studies, the tests of model fit for the research model were conducted using several fit indices, including the Comparative Fit Index (CFI), Tucker–Lewis Index (TLI), and Root Mean Square Error of Approximation (RMSEA). These indices were chosen for their ability to evaluate the model’s fit while considering its simplicity and suitability. The assessment of the proposed model’s fit yielded the following results: a CFI of 0.927, a TLI of 0.916, and an RMSEA of 0.077. According to the conventional thresholds, a CFI and TLI greater than 0.90 indicate a good fit, and an RMSEA less than 0.08 also signifies a good fit (Hu and Bentler, 1999; Kline, 2011). These results indicate that the proposed model fits well with the data, demonstrating both simplicity and suitability. Table 7 provides the detailed results of the model fit indices.

**Table 7:** Model fit.

	χ^2^	df	CFI	TLI	RMSEA
Fit	933.897	306	0.927	0.916	0.077

### Hypothesis verification

The path coefficients between the variables, as depicted in Table 8, are presented in accordance with the hypotheses of this study to verify their validity. H1: The analysis examining the relationship between functional value and attitude (PBT) estimated the path coefficient to be 0.174 (t = 2.148, *p* < 0.05), indicating that functional value has a significant positive effect on attitude. H2: The analysis identifying the relationship between emotional value and attitude (PBT) yielded a path coefficient of 0.126 (*t* = 1.685, *p* = 0.092), suggesting that emotional value does not significantly affect attitude. H3: The analysis identifying the relationship between social value and attitude (PBT) produced a path coefficient of 0.364 (*t* = 3.900, *p* < 0.001), indicating that social value has a significant positive effect on attitude. H4: The analysis identifying the relationship between attitude and behavioral intention estimated the path coefficient to be 0.192 (*t* = 3.576, *p* < 0.001), indicating that attitude positively affects behavioral intention. H5: The analysis of the relationship between subjective norms and behavioral intention found a path coefficient of 0.199 (*t* = 3.185, *p* < 0.001), indicating that subjective norms have a statistically significant effect on behavioral intention. H6: The analysis identifying the relationship between perceived behavioral control and behavioral intention estimated the path coefficient to be 0.127 (*t* = 1.981, *p* < 0.05), indicating that perceived behavioral control has a significant positive effect on behavioral intention. These results provide support for most of the hypotheses, demonstrating significant relationships between the majority of the constructs examined in this study. The only exception was the hypothesis regarding the relationship between emotional value and attitude, which did not reach statistical significance.

**Table 8:** Hypothesis test by structural parameter estimates.

	Factors	Path (β)	S.E.	*t*	Results
H1	Functional value → Attitudes	0.174	0.092	2.148*	Accepted
H2	Emotional value → Attitudes	0.126	0.072	1.685	Verified
H3	Social value→ Attitudes	0.364	0.070	3.900***	Accepted
H4	Attitudes → behavioral intention	0.192	0.054	3.576***	Accepted
H5	Subjective Norms → behavioral intention	0.199	0.061	3.185***	Accepted
H6	perceived behavioral control → behavioral intention	0.127	0.067	1.981*	Accepted

***p < 0.001; *p < 0.5.

## Discussion

### Theoretical implications

This study attempted to predict the behavior of Pilates participants based on the perceived value related to the formation process of Pilates participation intention. The results showed that among the three subfactors of perceived value, functional and social values positively affect attitudes toward Pilates, while emotional value does not have a significant effect. Additionally, subjective norms and perceived behavioral control positively affected Pilates behavioral intention.

First, the analysis of the relationships between functional, emotional, and social values and attitudes revealed that functional and social values positively influence the formation of attitudes toward Pilates among participants, but emotional value does not. The decision to regard functional and social values exclusively as determinants of attitude, instead of subjective norms or perceived behavioral control, is based on theoretical rationale. Previous studies indicate that perceived value predominantly affects an individual’s evaluative judgment (i.e., attitude) on an activity, as it embodies own evaluations of advantages and compromises rather than external influences or perceived limitations (Ajzen, 1991; Sweeney and Soutar, 2001). This internal appraisal process differentiates attitude from subjective norms and perceived behavioral control, which are more affected by external social and environmental influences. Consequently, value functions as a fundamental precursor of attitude, bolstering its influence on behavioral intention within the TPB framework.

Functional value impacts attitudes because it encompasses the practical benefits participants perceive from Pilates, such as improved physical health, strength, and flexibility. These tangible outcomes are directly aligned with the participants’ initial motivations for engaging in Pilates. The finding of this study aligns with prior research suggesting that functional value significantly influences attitude formation. Um and Yoon (2021) demonstrated that perceived functional value of a tourism gentrification experience positively shaped their attitudes, reinforcing the idea that tangible benefits play a crucial role in shaping evaluative judgments. Social value, on the other hand, had a more substantial impact on attitudes compared to functional value. This indicates that the improvement of interpersonal relationships, economic benefits, and time efficiency are significant factors in shaping positive attitudes towards Pilates. The community and social interactions experienced during Pilates classes contribute to a sense of belonging and support, which enhances participants’ overall satisfaction and commitment to the practice. The finding of this study is consistent with previous research demonstrating that social value significantly influences attitudes. Roh et al. (2022) found that green perceived value, encompassing social value, had a substantial impact on consumer attitudes toward organic food consumption, reinforcing the role of social factors in shaping evaluative judgments. Emotional value, however, did not significantly impact attitudes, which contrasts with other research emphasizing the importance of emotions in influencing attitudes (Roh et al., 2022). A potential reason for this divergence is that Pilates participants may emphasize concrete benefits, such as physical enhancement and social interaction, over emotional satisfaction when developing attitudes toward participation. In contrast to group-based fitness programs like Zumba or dance aerobics, which rely on emotional engagement as a primary motivator, Pilates prioritizes precision and control. This emphasis on skill and organized movement patterns may cause individuals to view functional and social advantages as more significant in influencing their attitudes than emotional considerations.

These findings contribute to the broader literature by validating the role of perceived value within the extended TPB framework. In contrast to conventional fitness pursuits, where emotional involvement greatly affects attitudes, Pilates participants seem to be primarily motivated by pragmatic and social advantages. This observation highlights the necessity for customized engagement tactics that correspond with the distinct attributes of Pilates practice.

Second, with respect to the prediction about the formation process of Pilates behavioral intention of Pilates participants, the analysis results of relationships between attitude, subjective norms, perceived behavioral control, and behavioral intention revealed that attitude, subjective norms, and perceived behavioral control all have a positive impact on behavioral intention. These results are consistent with studies such as those on the purchase intention for green products among Tai consumers by Maichum et al. (2016), which reported that attitude, subjective norms, and perceived behavioral control all positively affect purchase intention. Similarly, Lee (2012) reported the positive effects of TPB variables on participation in sports among the physically disabled, and Jang and Kim (2019) found that these variables positively affect the behavioral intention for continuous exercise participation among college students.

However, subjective norms were found to have the most substantial influence on behavioral intention, which is contrary to many previous sports studies where attitude was identified as the most critical factor (Seonwoo and Jeong, 2021). The influence of subjective norms indicates that the opinions and behaviors of significant others, such as instructors, peers, and social groups, play a crucial role in shaping participants’ intentions to continue Pilates. This finding suggests that social influence and the desire to meet the expectations of important others are strong motivators for continued engagement (Kim and Jeong, 2024; Yu and Jeong, 2022). Perceived behavioral control also significantly impacts behavioral intention as it reflects the participants’ confidence in their ability to perform Pilates exercises and overcome potential barriers. When participants feel capable and supported, they are more likely to intend to continue. Therefore, improving perceived behavioral control can involve ensuring that the Pilates environment, including location, equipment, amenities, and transportation, is conducive to participation.

These results confirm the robustness of the extended TPB model in explaining Pilates participation behavior. This study illustrates the preeminent role of social factors in determining behavioral intention, hence contesting traditional views that see attitude as the foremost determinant of intention. This discovery significantly contributes to exercise psychology by highlighting the impact of external factors on long-term participation behaviors.

Therefore, the findings of this study have significant academic implications. They suggest that in the context of Pilates, social and functional values play a more crucial role in shaping attitudes than previously thought, with social values being particularly influential. This highlights the need for further research into the social dimensions of fitness activities and their impact on participation. Moreover, the strong influence of subjective norms on behavioral intention challenges the traditional emphasis on attitudes as the primary predictor. This underscores the importance of considering social influences and perceived support systems in studies of exercise behavior. These insights contribute to the broader understanding of how different values and social factors interact to shape health-related behaviors, offering a more nuanced view of the factors driving Pilates participation. By recognizing the significant role of social influences, future research can better address the complex interplay of factors that motivate individuals to engage in and sustain fitness activities like Pilates.

### Practical implications

To promote the continuous growth of the Pilates industry, Pilates studios should invest in developing differentiated programs that reflect the emotional and sensory dimensions of participants. This will ensure that Pilates participation is perceived as enjoyable and attractive. Exercise programs with inventive elements that stimulate students’ interest are likely to be more effective than typical, indistinctive programs.

Enhancing social value within Pilates studios can be achieved by fostering a sense of community and encouraging social interactions. This can be done through organizing social events, group classes, and workshops that allow participants to connect and build relationships. Creating an environment where participants feel a sense of belonging and support can significantly enhance their commitment and enjoyment of Pilates.

Instructor training is another critical area for improvement. Ensuring that instructors are well-trained to provide positive reinforcement, build strong relationships with participants, and create an encouraging atmosphere can enhance participants’ perceived behavioral control and foster positive attitudes towards Pilates.

Improving the physical environment of Pilates studios is also essential. Studios should be easily accessible, well-equipped, and provide amenities such as comfortable changing rooms and relaxation areas. Addressing logistical barriers and ensuring a high-quality environment can improve overall participant satisfaction and encourage continued participation.

Promotional campaigns should highlight the functional and social benefits of Pilates. Emphasizing how Pilates can improve physical health, enhance social connections, and provide a valuable use of time can attract more participants and reinforce the benefits of continued participation.

Finally, implementing regular feedback systems to gather participants’ opinions and suggestions can help studios continually improve their programs and services. By responding to the evolving needs and preferences of participants, studios can ensure they remain relevant and engaging, ultimately supporting long-term retention and satisfaction.

## Conclusion

This study analyzed the behavioral intention of Pilates participants by applying an ETPB model, incorporating perceived value (functional, social, and emotional value) as an additional factor in the existing TPB model. The results suggested that the behavioral intention of Pilates participants can be improved through attitudes towards Pilates exercise and perceived behavioral control, both of which are also influenced by subjective norms. In addition, the perceived value significantly impacts both behavioral attitude and behavioral intention.

The study revealed that social value has a more substantial impact on attitudes compared to functional value. This finding indicates that the improvement of interpersonal relationships, economic benefits, and time efficiency are significant factors in shaping positive attitudes towards Pilates. The community and social interactions experienced during Pilates classes contribute to a sense of belonging and support, enhancing participants’ overall satisfaction and commitment to the practice. On the other hand, emotional value did not significantly influence attitudes, possibly because the immediate and measurable benefits of functional and social values overshadow the more subjective and personal aspects of emotional experiences. Participants might prioritize practical and social outcomes over emotional ones when evaluating their attitudes towards Pilates.

The analysis also showed that subjective norms have the most substantial influence on behavioral intention, which contrasts with many previous sports studies where attitude was identified as the most critical factor. This suggests that the opinions and behaviors of significant others, such as instructors, peers, and social groups, play a crucial role in shaping participants’ intentions to continue Pilates. Social influence and the desire to meet the expectations of important others are strong motivators for continued engagement. Perceived behavioral control also significantly impacts behavioral intention, reflecting the participants’ confidence in their ability to perform Pilates exercises and overcome potential barriers. When participants feel capable and supported, they are more likely to intend to continue.

To improve the behavioral intention of Pilates participants, efforts should focus on preventing the reduction of positive attitudes and enhancing perceived behavioral control for Pilates activities. It is also necessary to elevate the functional value of Pilates exercises and promote the formation of active social value accompanying participation in Pilates. Holding various events to induce participation, such as programs linked to social clubs of people with shared hobbies or interests, can be effective. Providing up-to-date information on facilities, locations, and participation effects through the homepage and various media can also increase perceived value.

Concerning the limitations of this study, although the perceived value had already been formed to some extent and affected the performance of Pilates behavior among participants, this study did not conduct a comparative analysis of perceived value considering the participants’ length of experience with Pilates. Future research should include a comparative analysis considering this factor to provide more elaborate interpretations and suggestions. Additionally, various explanatory variables reflecting motivation should be employed in future analyses of behavioral intention by extending the TPB. This approach will provide a broader understanding of the behavioral intention of Pilates participants and produce meaningful research outcomes that can be actively applied in the field. Moreover, future research could incorporate a mean comparison test to examine potential differences across demographic groups, such as gender and career. Analyzing whether these demographic factors influence perceived value and behavioral intention could provide deeper insights into individual variations in Pilates participation. Furthermore, future research should consider comparing Pilates practitioners with non-practitioners to determine whether the observed relationships hold across different groups. This comparison may offer important new information about the variables affecting both initial and ongoing involvement. Additionally, exploring the potential mediating impact of functional and social values on the relationship among attitude, subjective norms, perceived behavioral control, and behavioral intention may augment the model’s explanatory capacity. This method may elucidate the indirect mechanisms by which perceived value influences behavioral outcomes. In addition, this study offers useful information into Pilates participation; nevertheless, the generalizability of the findings is constrained by the reliance on convenience sampling and data collecting from select Pilates centers in a restricted region. Further studies should incorporate a more heterogeneous sample from other geographic and cultural contexts to improve the external validity of the results. Finally, future study could further refine the model by incorporating demographic control variables to explore their potential moderating effects. However, given that the primary objective of this study was to validate the theoretical framework of the extended TPB model, the exclusion of control variables does not undermine the validity of the findings.

## Data Availability

The original contributions presented in the study are included in the article/supplementary material, further inquiries can be directed to the corresponding author.

## References

[ref1] Ahmed I. Alzahrani I. M. Ramayah T. Alfarraj O. Alalwan N. (2017). Extending the theory of planned behavior (TPB) to explain online game playing among Malaysian undergraduate students. Telematics Inform. 34, 239–251. doi: 10.1016/j.tele.2016.07.001

[ref2] Ajzen I. (1991). The theory of planned behavior. Organ. Behav. Hum. Decis. Process. 50, 179–211. doi: 10.1016/0749-5978(91)90020-T

[ref3] Ajzen I. (2001). Nature and operation of attitudes. Annu. Rev. Psychol. 52, 27–58. doi: 10.1146/annurev.psych.52.1.27, PMID: 11148298

[ref4] Ajzen I. Driver B. L. (1992). Application of the theory of planned behavior to leisure choice. J. Leis. Res. 24, 207–224. doi: 10.1080/00222216.1992.11969889

[ref5] Ajzen I. Fishbein M. (2000). Attitudes and the attitude-behavior relation: reasoned and automatic processes. Eur. Rev. Soc. Psychol. 11, 1–33. doi: 10.1080/14792779943000116

[ref6] Armitage C. J. Conner M. (2001). Efficacy of the theory of planned behavior: a meta-analytic review. Br. J. Soc. Psychol. 40, 471–499. doi: 10.1348/014466601164939, PMID: 11795063

[ref8] Bae S. H. Noh J. H. Kang H. J. (2015). A study for the effect about intention of participation in medical tourism to Korea by using theory of planned behavior (TPB): focusing on the potential medical tourist in Japan. J. Tour. Sci. 39, 165–183. doi: 10.17086/JTS.2015.39.3.165.183

[ref9] Bagozzi R. (1981). Attitudes, intentions, and behavior: a test of some key hypotheses. J. Pers. Soc. Psychol. 41, 607–627. doi: 10.1037/0022-3514.41.4.607

[ref11] Cho S. H. (2019). A comparative study on the structural equation model between theory of planned behavior (TPB) and extended planned behavior theory (ETPB): focused on green marketing of coffee shop in Daejeon. J. Hosp. Tour. Stud. 21, 155–171. doi: 10.31667/jhts.2019.2.78.155

[ref12] Dawkins C. E. Frass J. W. (2005). Decision of union workers to participate in employee involvement: an application of the theory of planned behavior. Empl. Relat. 27, 511–531. doi: 10.1108/01425450510612031

[ref13] Fishbein M. Ajzen I. (1975). Belief, attitude, intention, and behavior: An introduction to theory and research. Reading, MA: Addison-Wesley.

[ref14] Ha J. Kim S. (2023). Effects of perceived value on sustainable behavioral intention among Pilates participants: focusing on the extended theory of planned behavior (ETPB) [Preprint].

[ref9001] Hagger M. S. Wood C. Stiff C. Chatzisarantis N. L. D. (2010). Ego depletion and the strength model of self-control: A meta-analysis. Psychol. Bull. 136, 495–525. doi: 10.1037/a0019486 20565167

[ref9002] Han S. Lerner J. S. Keltner D. (2014). Feelings and consumer decision making: The appraisal-tendency framework. J. Consum. Psychol. 24, 2–19. doi: 10.1016/S1057-7408(07)70023-2

[ref15] Hair J. F. Black W. C. Babin B. J. Anderson R. E. (2010). Multivariate data analysis: A global perspective. 7th Edn. Upper Saddle River, NJ: Pearson Prentice Hall.

[ref16] Hausenblas H. A. Carron A. V. Marck D. E. (1997). Application of the theories of reasoned action and planned behavior to exercise behavior: a meta-analysis. J. Sport Exerc. Psychol. 19, 36–51. doi: 10.1123/jsep.19.1.36

[ref17] Henseler J. Ringle C. M. Sarstedt M. (2015). A new criterion for assessing discriminant validity in variance-based structural equation modeling. J. Acad. Mark. Sci. 43, 115–135. doi: 10.1007/s11747-014-0403-8

[ref18] Holbrook M. B. (1999). Consumer value: A framework for analysis and research. New York: Routledge.

[ref20] Hu L. T. Bentler P. (1999). Cutoff criteria for fit indexes in covariance structure analysis: conventional criteria versus new alternatives. Struct. Equ. Model. Multidiscip. J. 6, 1–55. doi: 10.1080/10705519909540118

[ref21] Jamal S. A. Muhammad N. M. N. (2011). Tourist perceived value in a community-based home stay visit: an investigation into the functional and experiential aspects of value. J. Vacat. Mark. 17, 5–15. doi: 10.1177/1356766710391130

[ref22] Jang B. Kim S. (2019). A structural relationships between college athletes’ perceived coaching behaviors, affective responses, emotional intelligence, and performance. Korean Soc. Sport Psychol. 30, 1–13. doi: 10.14385/KSSP.30.2.1

[ref24] Joseph E. M. Simona C. (2004). Pilates and the “powerhouse” – II. J. Bodyw. Mov. Ther. 8, 122–130. doi: 10.1016/S1360-8592(03)00064-4

[ref29] Kim D. H. Jeong Y. (2024). Examining the moderating effect of perceived risk from particulate matter on outdoor sports participants: a theory of planned behavior perspective. Front. Public Health 12:1340502. doi: 10.3389/fpubh.2024.1340502, PMID: 38344237 PMC10853401

[ref27] Kim G. A. (2014). Study on the relationship between perceived value, attitude and behavioral intention of fair tourism: focus on tourist’s experience in the Gamcheon Cultural Village. J. Cult. Policy 28, 174–197. doi: 10.16937/jcp.28.2.201408.174

[ref31] Kim J. O. Moon B. Y. (2015). The influence of auto-camper’s value on attitude and behavioral intention: an application of the extended theory of planned behavior. J. Tour. Leisure Res. 27, 39–58. doi: 10.31336/jtlr.2015.27.1.39

[ref32] Kim S. J. Ryu D. S. (2016). The relationships between values, brand attitude and intended action in university Tae-Kwon-do competition. Korea Contents Assoc. 16, 464–476. doi: 10.5392/JKCA.2016.16.11.464

[ref28] Kim Y. Jeong Y. (2022). Exploring the structural relationships between pilates instructors’ expertise, trust, coaching effectiveness and participants’ intention to continue pilates. J. Sport Leisure Stud. 90, 295–308. doi: 10.51979/KSSLS.2022.10.90.295

[ref30] Kim Y. J. Kim E. S. (2021). Analysis of Korean fencing club members’ participation intention using the TPB model. Int. J. Environ. Res. Public Health 18:2813. doi: 10.3390/ijerph18062813, PMID: 33802048 PMC8002150

[ref33] Kline R. B. (2011). Principles and practice of structural equation modeling. New York: Guilford Press.

[ref34] Kwon H. Kim D. Shin J. E. (2017). Fitness participation intention and the actual participation behavior predicted by the theory of planned behavior. Korean J. Sport Manag. 22, 55–69. doi: 10.31308/KSSM.22.3.4

[ref35] Lam T. Hsu C. H. C. (2004). Theory of planned behavior: potential travelers from China. J. Hosp. Tour. Res. 28, 463–482. doi: 10.1177/1096348004267515

[ref36] Lee H. S. (2012). Analysis of model of sport for all participation behavior with people with physical disabilities applied to the TPB. Korean J. Phys. Educ 51, 431–440.

[ref38] Lin L. Y. Chen C. S. (2006). The influence of the country-of-origin image, product knowledge, and product involvement on consumer purchase decisions: an empirical study of insurance and catering services in Taiwan. J. Consum. Mark. 23, 248–265. doi: 10.1108/07363760610681655

[ref39] Lu Y. J. Lai H. R. Lin P. C. Kuo S. Y. Chen S. R. Lee P. H. (2022). Predicting exercise behaviors and intentions of Taiwanese urban high school students using the theory of planned behavior. J. Pediatr. Nurs. 62, e39–e44. doi: 10.1016/j.pedn.2021.07.001, PMID: 34272134

[ref9300] Maichum K. Parichatnon S. Peng K.-C. (2016). Application of the extended theory of planned behavior model to investigate purchase intention of green products among Thai consumers. Sustain 8:1077. doi: 10.3390/su8101077

[ref41] Ouellette J. A. Wood W. (1998). Habit and intention in everyday life: the multiple processes by which past behavior predicts future behavior. Psychol. Bull. 124, 54–74. doi: 10.1037/0033-2909.124.1.54

[ref44] Quintal M. V. Lee J. Soutar G. (2010). Risk, uncertainty, and the TPB: a tourism example. Tour. Manag. 31, 797–805. doi: 10.1016/j.tourman.2009.08.006

[ref45] Roh T. Seok J. Kim Y. (2022). Unveiling ways to reach organic purchase: green perceived value, perceived knowledge, attitude, subjective norm, and trust. J. Retail. Consum. Serv. 67:102988. doi: 10.1016/j.jretconser.2022.102988

[ref46] Seo H. R. Lee C. G. (2015). A study of Chinese tourists’ decision-making process in visiting South Korea using extended theory of planned behavior. J. Hosp. Tour. Stud. 17, 1–16.

[ref48] Seonwoo Y. Y. Jeong Y. D. (2021). Exploring factors that influence taekwondo student athletes’ intentions to pursue careers contributing to the sustainability of the Korean taekwondo industry using the theory of planned behavior. Sustain. For. 13:9893. doi: 10.3390/su13179893

[ref47] Seo Y. H. Moon H. Lee J. E. Shin J. H. Jeong H. J. Kim K. (2015). The prediction of participating in continuous exercise applying extended the theory of planned behavior: focus on participating students in university physical education class. Korean J. Sports Sci. 24, 211–226.

[ref49] Sheth J. N. Newman B. I. Gross B. L. (1991). Why we buy what we buy: a theory of consumption values. J. Bus. Res. 22, 159–170. doi: 10.1016/0148-2963(91)90050-8

[ref50] So Y. H. (2021). Analysis on behavioral intention of marine sports participants by using extended theory of planned behavior. Korean J. Phys. Educ. 60, 371–385. doi: 10.23949/kjpe.2021.1.60.1.27

[ref51] Sweeney J. C. Soutar G. N. (2001). Consumer perceived value: the development of a multiple item scale. J. Retail. 77, 203–220. doi: 10.1016/S0022-4359(01)00041-0

[ref52] Sweeney J. C. Soutar G. N. Johnson L. W. (1999). The role of perceived risk in the quality-value relationship: a study in a retail environment. J. Retail. 75, 77–105. doi: 10.1016/S0022-4359(99)80005-0

[ref53] Taylor S. Todd P. A. (1995). Understanding information technology usage: a test of competing models. Inf. Syst. Res. 6, 144–176. doi: 10.1287/isre.6.2.144, PMID: 19642375

[ref54] Teng Y. M. Wu K. S. Huang D. M. (2014). The influence of green restaurant decision formation using the VAB model: the effect of environmental concern upon intent to visit. Sustain. For. 6, 8736–8755. doi: 10.3390/su6128736

[ref55] Thompson C. G. Kim R. S. Aloe A. M. Becker B. J. (2017). Extracting the variance inflation factor and other multicollinearity diagnostics from typical regression results. Basic Appl. Soc. Psychol. 39, 81–90. doi: 10.1080/01973533.2016.1277529

[ref57] Um J. Yoon S. (2021). Evaluating the relationship between perceived value regarding tourism gentrification experience, attitude, and responsible tourism intention. J. Tour. Cult. Chang. 19, 345–361. doi: 10.1080/14766825.2019.1707217

[ref58] Vallerand R. J. Pelletier L. G. Blais M. R. Brière N. M. Senècal C. Vallières E. F. (1993). On the assessment of intrinsic, extrinsic, and amotivation in education: evidence on the concurrent and construct validity of the academic motivation scale. Educ. Psychol. Meas. 53, 159–172. doi: 10.1177/0013164493053001018

[ref60] Yoon S. M. Oh S. Y. Ha J. Y. (2011). Analysis for effect relationship among perceived value, satisfaction, attitude, and behavioral intention of visitor in local festival: the case of Andong mask dance festival visitors. J. Hosp. Tour. Stud. 13, 82–97.

[ref61] Yoon S. M. Oh S. Y. Yoon S. J. (2010). A study for the effect relationship about overseas trip intention of local by using theory of planned behavior (TPB): focusing on the additional role of prior knowledge and perceived risk. J. Hosp. Tour. Stud. 19, 289–307.

[ref62] Yu J. G. Jeong Y. (2022). A case study of factors impacting aspiring E-sports athletes in South Korea. Studia Sport. 16, 214–228. doi: 10.5817/StS2022-2-22

[ref63] Zeithaml V. A. (1988). Consumer perceptions of price, quality, and value: a means-end model and synthesis of evidence. J. Mark. 52, 2–22. doi: 10.1177/002224298805200302

[ref65] Zhang W. J. Xu M. Feng Y. J. Mao Z. X. Yan Z. Y. Fan T. F. (2022). The value-added contribution of exercise commitment to college students’ exercise behavior: application of extended model of theory of planned behavior. Front. Psychol. 13:869997. doi: 10.3389/fpsyg.2022.869997, PMID: 35719512 PMC9204293

